# Knowledge of and willingness to use tobacco cessation services among patients with non-communicable diseases in Bangladesh: A cross-sectional study

**DOI:** 10.1371/journal.pone.0356915

**Published:** 2026-08-26

**Authors:** Md. Golam Kibria, Russell Kabir, Ali Davod Parsa, Mst. Nasrin Jebin, Sonali Akter, Md. Abu Raihan, Taslima Islam

**Affiliations:** 1 Department of Research, Centre for Development Action, Dhaka, Bangladesh; 2 School of Allied Health, Anglia Ruskin University, Chelmsford, Essex, United Kingdom; 3 Department of Health Service, Directorate General of Health Services, Dhaka, Bangladesh; 4 Department of Statistics, University of Rajshahi, Rajshahi, Bangladesh; 5 Department of Monitoring, Evaluation and Learning, Social Development Foundation, Dhaka, Bangladesh; University of Sydney, AUSTRALIA

## Abstract

**Background:**

Tobacco cessation services are recognised as an effective strategy for curbing tobacco consumption. However, data regarding these services in Bangladesh are limited. This study aimed to assess the levels of knowledge of and willingness to use tobacco cessation services and to identify associated factors among patients with NCDs who were current tobacco users in Bangladesh.

**Methods:**

A cross-sectional study was conducted among 660 consecutively selected patients with NCDs aged ≥18 years visiting selected government sub-district hospitals in Bangladesh. Data were collected using a structured questionnaire from May 26–14 June, 2025. Multivariable logistic regression analyses were performed to identify the factors associated with overall knowledge of and willingness to use cessation services.

**Results:**

Only 10.5% of participants reported moderate to high levels of overall knowledge on cessation services, while 65.3% expressed willingness to use them. In the multivariable ordinal logistic regression with partial proportional odds model (PPOM), higher knowledge of cessation services was associated with secondary education (adjusted odds ratio [aOR]: 1.9, 95% CI: 1.08–3.31) and higher secondary education and above (aOR: 4.3, 95% CI: 2.21–8.51). Similarly, the multivariable binary logistic regression analysis shows that willingness to use cessation services was associated with secondary education (aOR: 1.8, 95% CI: 1.12–2.83), higher secondary education and above (aOR: 2.2, 95% CI: 1.13–4.68), > 10 hospital visits (aOR: 2.5, 95% CI: 1.59–3.88), and moderate to high levels of overall knowledge of cessation services (aOR: 3.5, 95% CI: 1.73–7.99).

**Conclusions:**

Despite low levels of knowledge, willingness to use cessation services was high among participants in Bangladesh. Educational attainment was a key determinant of knowledge, while greater knowledge, higher education, and more frequent hospital visits were associated with willingness. These findings highlight the need to improve patient awareness and integrate cessation support into routine care to increase service use among tobacco users.

## 1 Introduction

Tobacco use is a well-established risk factor for a wide range of non-communicable diseases (NCDs), including cardiovascular disease, chronic respiratory illnesses, diabetes, and several forms of cancer [1,2]. Globally, tobacco use accounts for more than eight million deaths annually, with around 80% of tobacco users living in low- and middle-income countries (LMICs) [3,4]. In Bangladesh, 35.3% of adults aged 15 years and above use tobacco in either smoked or smokeless forms [5]. NCDs alone account for over 67% of all deaths nationwide, reflecting a substantial and growing public health burden [6]. The co-existence of high tobacco use and the rising prevalence of NCDs presents a major challenge to the country’s healthcare system.

Patients with NCDs constitute a priority population for tobacco cessation interventions. A diagnosis of an NCD can serve as a “teachable moment”, during which patients are more receptive to cessation advice and support from healthcare providers [7,8]. Research shows that tobacco cessation significantly reduces the risk of disease progression, complications, and premature death among patients with NCDs [9–12]. Evidence indicates that cessation improves clinical outcomes and quality of life [13,14], highlighting its critical role in the prevention and management of NCDs. Integrating tobacco cessation services into routine NCD care is widely recognised as an effective public health strategy.

Primary healthcare (PHC) facilities are well-positioned to provide tobacco cessation support, as they serve as the first points of contact with patients and offer repeated opportunities through routine consultations, follow-up visits, and chronic disease management [15,16]. In Bangladesh, government sub-district hospitals, commonly known as *upazila health complexes*, cater to a large proportion of patients with NCDs, particularly in rural and peri-urban areas. However, despite the adoption of several tobacco control policies aligned with the WHO Framework Convention on Tobacco Control (FCTC), cessation services remain inadequately integrated into the country’s public healthcare system [17].

In Bangladesh, cessation support in healthcare facilities is mainly limited to tobacco use screening and brief advice, with limited provision of structured behavioural counselling or pharmacological treatment [5,18,19]. This situation is largely driven by shortages of healthcare personnel, lack of training and knowledge, high workload and time constraints, and lack of national guidelines for cessation services [20]. Such structural barriersmay restrict patients’ access to and utilisation of cessation services, even when they are motivated to quit.

At the same time, individual-level factors also play a critical role in shaping service uptake. Patients’ knowledge of available cessation services and their willingness to use such services are critical determinants of engagement with cessation support. Even where cessation services are available, low awareness of cessation services, limited perceived benefits of quitting, social acceptability of tobacco use, and insufficient support from health professionals may reduce utilisation of cessation services [21–23]. Despite the importance of these factors, there is limited evidence on patients’ knowledge of and willingness to use cessation services in Bangladesh.

This study aims to assess the levels of knowledge of and willingness to use cessation services among patients with NCDs who were current tobacco users attending government sub-district hospitals in Bangladesh. By identifying gaps and associated factors, the findings will provide important insights to inform the design and integration of patient-centred tobacco cessation interventions within NCD care, thereby strengthening tobacco control efforts and improving population health outcomes in Bangladesh.

## 2 Methods

### 2.1 Study design and participants

This cross-sectional study was conducted among patients with NCDs who received services at NCD corners in Bangladesh. In 2012, the government initiated the establishment of one NCD corner in each sub-district hospital with the aim of providing preventive and curative services for common NCDs [24]. These dedicated units offer screening, diagnosis, treatment, counselling, and referral for chronic conditions such as diabetes mellitus, cardiovascular diseases, chronic respiratory illnesses, and cancers. This study recruited patients under the following criteria: (a) aged 18 years or older (b) daily users of smoked, smokeless, or both forms of tobacco for a minimum of five years, and (c) diagnosed with at least one of the following NCDs: Diabetes mellitus, asthma, chronic obstructive pulmonary disease (COPD), bronchitis, hypertension, heart disease, stroke, chronic kidney disease (CKD), and cancer. A duration of at least five years of tobacco use was set as an inclusion criterion, as individuals with prolonged tobacco exposure may have experienced significant tobacco-related health consequences that could influence their quitting behaviours. On the other hand, patients who did not use tobacco daily or experienced hearing and/or speech impairments that could hinder communications during data collection were excluded from this study. This manuscript adheres to the STROBE (Strengthening the Reporting of Observational Studies in Epidemiology) reporting guidelines for cross-sectional studies. For this study, ethical approval was obtained from the National Research Ethics Committee of the Bangladesh Medical Research Council (BMRC) on 25 March, 2025 [Ref: BMRC/NREC/2025–2027/116(1–195)].

### 2.2 Sampling and study setting

A multistage cluster sampling approach combining both probability and non-probability techniques was employed to recruit study participants. The sample size was calculated using the formula: n = $\frac{{\text{z}}^{\text{2}}\text{pq}}{{\text{e}}^{\text{2}}}$ [25], where z = 1.96 (95% confidence interval), p = 0.5 (assumed proportion of patients with NCDs having knowledge of cessation services in Bangladesh, and e = 0.05 (margin of error). After adjusting for a design effect of 1.5 [26] and a 10% non-response rate, the minimum required sample size was 640. To maximise statistical precision, recruitment continued until the end of the planned data collection period as eligible participants continued to attend the selected facilities. This resulted in a final sample of 660 participants.

**Fig 1:**
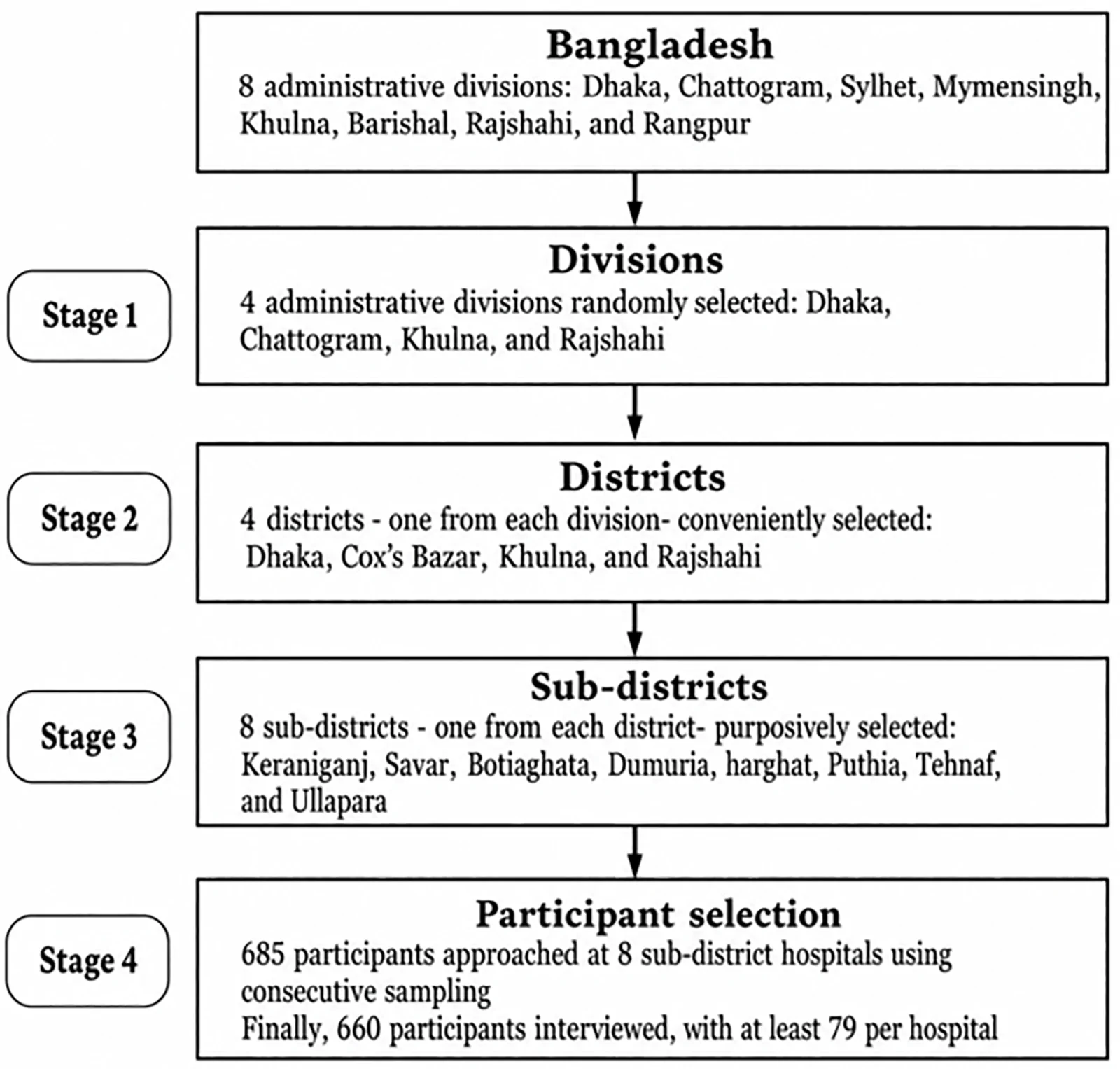
Flow chart of the multistage cluster sampling procedure for participant selection.

Bangladesh comprises 64 districts and 495 sub-districts, with 483 government sub-district hospitals, of which 435 have operational NCD corners [27,28]. These Upazila Health Complexes (UHCs) serve as the primary healthcare facilities at the sub-district level. The sampling procedure involved four stages, as illustrated in Fig 1. In the first stage, four of the eight administrative divisions (Dhaka, Chattogram, Khulna, and Rajshahi) were randomly selected to enhance geographic representativeness. In the second stage, one district from each division was chosen based on feasibility and logistical considerations (Dhaka, Cox’s Bazar, Khulna, and Rajshahi). In the third stage, eight sub-districts (upazilas) with government hospitals hosting functional NCD corners were purposively selected (Keraniganj, Savar, Botiaghata, Dumuria, Charghat, Puthia, Teknaf, and Ukhiya) to ensure the inclusion of facilities capable of providing NCD-related services. In the final stage, participants were recruited using consecutive sampling [29], whereby all eligible patients attending the selected facilities during the data collection period were approached. A total of 715 participants were approached during the study period, of whom 660 successfully completed the interview, yielding a response rate: 92.3% (660/715), with a minimum of 78 and a maximum of 92 participants per hospital. The remaining 55 eligible participants declined participation or were unable to complete the interview. Consecutive sampling was considered appropriate for this facility-based study, as it allowed systematic inclusion of all eligible patients attending routine care settings, thereby reducing selection bias within facilities and capturing a representative patient flow during the study period.

### 2.3 Study variables

#### 2.3.1 Outcome variables.

This study had two outcome variables: (a) overall knowledge of tobacco cessation services and (b) willingness to use tobacco cessation services. Patients’ overall knowledge of tobacco cessation services was assessed using a three-item composite scale developed for this study. First, participants were asked whether they had ever heard of services that help people quit tobacco (Yes/No). Second, those who answered affirmatively to the first item were asked to identify the specific types of cessation services they were aware of (multiple response). Service types included brief advice, counselling, nicotine gum, nicotine patch, lozenges, mouth spray, nasal spray, medicines, and quitline services. Third, participants who reported awareness of at least one cessation service were asked to indicate the sources from which these services could be obtained (multiple response). Response options included pharmacies, private clinics, government hospitals, online platforms, and other facilities. For scoring purposes, an affirmative response to the first item was assigned a value of 1 and a negative response a value of 0. For the second and third items, reporting awareness of at least one service type or service location was coded as 1, and 0 otherwise. Scores across the three items were summed to generate a composite knowledge score ranging from 0 to 3, with higher scores indicating greater knowledge of tobacco cessation services. For analytical purposes, the composite score was subsequently categorised into three levels: no knowledge (score = 0), low levels of knowledge (score = 1), and moderate to high levels of knowledge (scores = 2–3). However, this scale demonstrated excellent internal consistency (Cronbach’s alpha = 0.918).

Willingness to use tobacco cessation services was measured using a single-item question: “Are you willing to use any tobacco cessation service to help you quit?” Response options included Yes, May be, or No. For analytical purposes, responses were dichotomised, with “Yes” indicating willingness, and May be or No coded as “No” to represent lack of willingness.

#### 2.3.2 Explanatory variables.

This study included three categories of explanatory variables: demographic, clinical, and tobacco use-related characteristics. Demographic variables were sex, age, education level, marital status, occupational category, and monthly family income. As an occupational category, service holders referred to those who were currently or formerly engaged in service. Clinical variables comprised number of chronic diseases duration of chronic illness, and annual hospital visit frequency. Tobacco use-related characteristics included forms of tobacco, daily tobacco use frequency, time to first tobacco use of the day, and duration of tobacco use. Smoked tobacco included bidis and cigarettes, whereas smokeless tobacco comprised gul, zarda, and sada pata.

### 2.4 Data collection

Four teams, each comprising one male and one female data collector with a minimum of a bachelor’s degree in science or social science, were recruited. Data collectors received two days of training on study objectives and protocol, the survey questionnaire, interviewing techniques, ethical considerations, and procedures for handling inconsistent responses. Training methods included lectures, demonstrations, and role-plays. Data collectors’ competency was assessed through observed mock interviews with feedback from trainers and a short post-training evaluation. After completion of the training, a structured survey questionnaire was pretested among 20 patients with NCDs in similar settings and refined based on feedback from data collectors. In addition, completed interviews were reviewed to ensure consistency across data collectors; any discrepancies identified during review were discussed and resolved. Upon receiving formal approval from sub-district hospital authorities, each team was assigned to two hospitals. On scheduled data collection days, teams positioned themselves outside the NCD corners of the selected hospitals. Following patient consultations, data collectors approached individuals, explained the study, outlined eligibility criteria, and emphasised voluntary participation. Eligible participants were escorted to a private area to ensure privacy. Written informed consent was obtained from all literate participants. For participants who were unable to provide written consent due to illiteracy, verbal informed consent was taken in the presence of an impartial adult (≥18 years) witness, and the consent process was documented by the witness’s signature. The use of verbal consent and its documentation procedure were approved by the National Research Ethics Committee of the Bangladesh Medical Research Council (BMRC). Data were collected using a pretested, structured survey questionnaire (Supplementary File 1), deployed on KoboCollect, an Android-based data collection app (https://support.kobotoolbox.org/data_collection_kobocollect.html). The app was configured to require a response to each question before proceeding to the next. This approach minimised the risk of missing data during data collection. However, the principal investigator and the co-investigator visited the study sites during data collection to monitor data quality. Each interview lasted approximately 15 minutes. The questionnaire was developed in English, translated into Bengali, and back-translated to ensure accuracy. Data collection occurred between May 26 and 14 June, 2025. No financial incentives were provided to the study participants.

### 2.5 Statistical analysis

Data collected using KoboCollect were exported to Microsoft Excel and subsequently cleaned before analysis. Statistical analysis was performed using R statistical software version 4.4.2. This study included two outcome variables: *overall knowledge of tobacco cessation services* and *willingness to use tobacco cessation services*. All explanatory variables were categorical and were summarised using frequencies and percentages. Bivariate associations between the outcome variables and explanatory variables were determined using Pearson’s chi-square test; Fisher’s exact test was employed when any expected cell count was less than five [25]. Variables with a p-value <0.05 in the bivariate analysis were considered for inclusion in the multivariable model: sex, education level, occupational category, monthly family income, annual hospital visit frequency, and duration of tobacco use. Given the ordinal nature of the outcome variable, *overall knowledge of tobacco cessation services* (no, low, and moderate/high), ordinal logistic regression was selected. Multicollinearity was assessed using the variance inflation factor (VIF), with all values below the threshold of 5 [30]. The proportional odds (PO) assumption was tested using the likelihood ratio test of nominal effects and was satisfied for all covariates except for annual hospital visit frequency (p = 0.017). Therefore, a partial proportional odds model (PPOM) with logit function was fitted to relax the PO assumption for annual hospital visit frequency, while maintaining it for other variables [31].

The second outcome variable, *willingness to use tobacco cessation services,* was binary (yes or no). Multivariable binary logistic regression was performed to examine the factors associated with willingness to use tobacco cessation services. Variables that showed a p-value < 0.05 in the bivariate analysis were included for the multivariable binary logistic model, namely sex, education level, occupational category, annual hospital visit frequency, daily tobacco use frequency, and overall knowledge of cessation services. Model fit was assessed using the Hosmer-Lemeshow test, which indicated adequate fit (p > 0.05) [32]. Multicollinearity was checked using the variance inflation factor (VIF), with all variables demonstrating VIF values below 5, indicating the absence of multicollinearity [30]. Results from both the partial proportional model and the binary logistic regression model are reported as adjusted odds ratios (AORs) with 95% confidence intervals (CIs). All analyses used two-sided tests with a significance level of 0.05.

## 3 Results

### 3.1 Demographic, clinical, and tobacco use characteristics

Table 1 presents the demographic, clinical, and tobacco use characteristics of study participants. Of the 660 participants, the majority were female (63.0%), aged 40–59 years (52.1%), and either housewives or unemployed (65.2%). Hypertension was the most prevalent chronic condition (57.7%), followed by diabetes mellitus (53.5%). Most participants reported having one chronic disease (51.1%), a disease duration of 5–10 years, and exclusive use of smokeless tobacco (75.5%).

**Table 1:** Demographic, clinical, and tobacco use characteristics of study participants.

Variable	Category	Frequency	Percentage
**Sex**	Male	244	37.0%
	Female	416	63.0%
**Age**	<40 years	68	10.3%
	40 to 59 years	344	52.1%
	≥60 years	248	37.6%
**Education level**	Illiterate/Primary	465	70.5%
	Secondary	129	19.5%
	Higher secondary and above	66	10.0%
**Marital status**	Married	581	88.0%
	Other (single and widowed)	79	12.0%
**Occupational category**	Housewife/Unemployed	420	63.6%
	Businessperson	75	11.4%
	Service holder	46	7.0%
	Manual worker	119	18.0%
**Monthly family income**	<20,000 BDT	317	48.0%
	20,000–30,000 BDT	270	40.9%
	>30,000 BDT	73	11.1%
^**a**^ **Chronic disease**	Asthma	94	14.2%
	Bronchitis	28	4.2%
	COPD	43	6.5%
	Diabetes mellitus	353	53.5%
	Hypertension	381	57.7%
	Heart disease	81	12.3%
	Stroke	40	6.1%
	CKD	27	4.1%
**Number of chronic diseases**	1 disease	337	51.1%
	2 diseases	267	40.5%
	≥3 diseases	56	8.5%
**Duration of chronic illness**	<5 years	259	39.2%
	5 to 10 years	313	47.4%
	>10 years	88	13.3%
**Annual hospital visit frequency**	1 to 5 times	358	54.2%
	6 to 10 times	119	18.0%
	>10 times	183	27.7%
**Forms of tobacco**	Smoked tobacco	80	12.1%
	Smokeless tobacco	498	75.5%
	Both	82	12.4%
**Daily tobacco use frequency**	1 to 5 times	411	62.3%
	6 to 10 times	152	23.0%
	>10 times	97	14.7%
**Time to first tobacco use of the day**	Up to 30 minutes after waking up	165	25.0%
	31 to 60 minutes after waking up	191	28.9%
	Beyond 60 minutes after waking up	304	46.1%
**Duration of tobacco use**	≤10 years	130	19.7%
	11 to 20 years	171	25.9%
	>20 years	359	54.4%

*Notes*: ^a^Frequencies and percentages were calculated using a multiple response analysis, with percentages expressed relative to the total number of cases; BDT, Bangladeshi taka; COPD, Chronic obstructive pulmonary disease; CKD, Chronic kidney disease.

### 3.2 Item-specific knowledge of cessation services

Table 2 presents participants’ knowledge of specific cessation service items. Only 13.5% of participants reported ever having heard of cessation services. Among those who were aware of such services, brief advice was the most commonly reported type (77.9%), followed by counselling (48.5%). Interestingly, awareness of pharmacological options was very low among participants. In terms of service delivery facilities, about three-fourths (74.6%) of participants mentioned private hospitals as a source of cessation services, while 41.3% reported government hospitals.

**Table 2:** Item-specific knowledge of tobacco cessation services, types of services, and service facilities among study participants.

Knowledge item	Category	Frequency	Percentage
Ever heard of cessation services (N = 660)	Yes	89	13.5%
	No	571	86.5%
Types of cessation services heard of (N = 68)*	Brief advice	53	77.9%
	Counselling	33	48.5%
	Nicotine gum	5	7.4%
	Nicotine patch	13	19.1%
	Nicotine lozenge	6	8.8%
	Mouth spray	5	7.4%
	Cessation medication	9	13.2%
	Other services	5	7.4%
Awareness of cessation service facilities (N = 63)*	Pharmacy	9	14.3%
	Private hospital	47	74.6%
	Government hospital	26	41.3%
	NGO health centre	17	27.0%
	Online support	14	22.2%

* Frequencies and percentages were calculated using a multiple response analysis, with percentages expressed relative to the total number of cases; NGO, Non-government organisation.

### 3.3 Overall knowledge of and willingness to use cessation services

**Fig 2:**
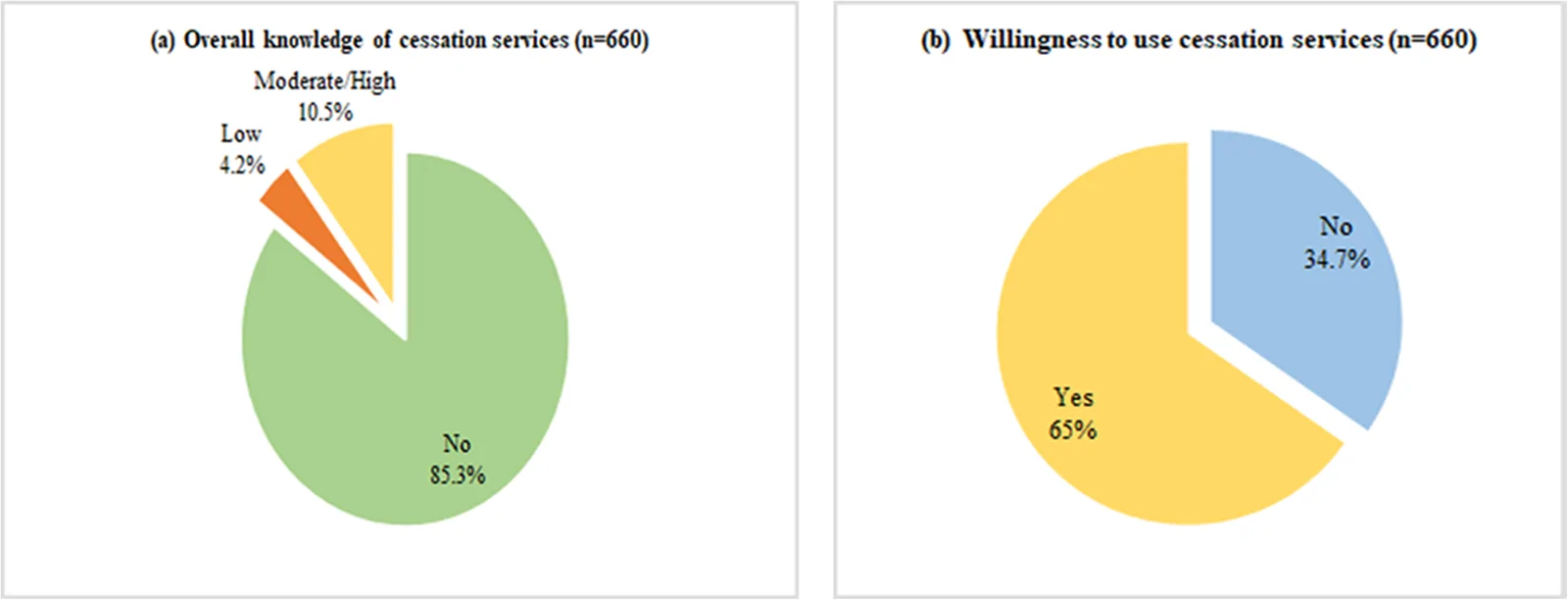
Distribution of overall knowledge and willingness to use cessation services among participants.

Fig 2 shows the distribution of overall knowledge of and willingness to use cessation services among study participants. Of the 660 participants, only 10.5% reported moderate to high levels of overall knowledge of cessation services. On the other hand, nearly two-thirds (65.3%) of participants showed willingness to use cessation services.

### 3.4 Bivariable associations with overall knowledge of and willingness to use cessation services

**Table 3** illustrates the bivariable associations between participants’ demographic, clinical, and tobacco use characteristic and their overall knowledge of and willingness to use cessation services. Overall knowledge was significantly associated with sex (p = 0.011), education level (p < 0.001), occupational category (p = 0.001), monthly family income (p = 0.021), annual hospital visit frequency (p = 010), and duration of tobacco use (p = 0.026). Similarly, willingness to use cessation services had significant associations with sex (p = 0.006), education level (p = .0.001), occupational category (p = 0.001), annual hospital visit frequency (p < 0.001), daily tobacco use frequency (p = 0.004), and overall knowledge of cessation services (p < 0.001).

**Table 3:** Bivariable analysis of overall knowledge of and willingness to use cessation among study participants by demographic, clinical, and tobacco use characteristics.

Variable	Overall knowledge of cessation services				Willingness to use cessation services		
	No	Low	Moderate/High	p-value^†^	Yes	No	p-value
	n (%)	n (%)	n (%)		n (%)	n (%)	
**Sex**							
Male	195 (79.9)	15 (6.1)	34 (13.9)	**0.011**	143 (58.6)	101 (41.4)	**0.006**
Female	368 (88.5)	13 (3.1)	35 (8.4)		288 (69.2)	128 (30.8)	
**Age**							
<40 years	53 (77.9)	3 (4.4)	12 (17.6)	0.244^‡^	50 (73.5)	18 (26.5)	0.290
40 to 59 years	298 (86.6)	17 (4.9)	29 (8.4)		224 (65.1)	120 (34.9)	
≥60 years	214 (86.3)	8 (3.2)	26 (10.5)		157 (63.3)	91 (36.7)	
**Education level**							
Illiterate/Primary	416 (89.5)	15 (3.2)	34 (7.3)	**<0.001**	283 (60.9)	182 (39.1)	**0.001**
Secondary	105 (81.4)	6 (4.7)	18 (14.0)		96 (74.4)	33 (25.6)	
Higher secondary and above	42 (63.6)	7 (10.6)	17 (25.8)		52 (78.8)	14 (21.2)	
**Marital status**							
Married	496 (85.4)	25 (4.3)	60 (10.3)	0.963^‡^	376 (64.7)	205 (35.3)	0.390
Other (single and widowed)	67 (84.8)	3 (3.8)	9 (11.4)		55 (69.6)	24 (30.4)	
**Occupational category**							
Housewife/Unemployed	370 (88.1)	14 (3.3)	36 (8.6)	**0.001** ^‡^	295 (70.2)	134 (29.8)	**0.001**
Businessperson	54 (73.3)	7 (9.3)	14 (18.7)		45 (60.0)	30 (40.0)	
Service holder	33 (71.7)	3 (6.5)	10 (21.7)		30 (65.2)	16 (34.8)	
Manual worker	106 (89.1)	4 (3.4)	9 (7.6)		61 (51.3)	58 (48.7)	
**Monthly family income**							
<20,000 BDT	279 (88.0)	11 (3.5)	27 (8.5)	**0.021**	203 (64.0)	114 (36.0)	0.374
20,000–30,000 BDT	231 (85.6)	12 (4.4)	27 (10.0)		175 (64.8)	95 (35.2)	
>30,000 BDT	53 (72.6)	5 (6.8)	15 (20.5)		53 (72.6)	20 (27.4)	
**Number of chronic diseases**							
1 disease	291 (86.4)	13 (3.9)	33 (9.8)	0.761^‡^	231 (68.5)	106 (31.5)	0.198
2 diseases	227 (85.0)	12 (4.5)	28 (10.5)		166 (62.2)	101 (37.8)	
≥3 diseases	45 (80.4)	3 (5.4)	8 (14.3)		34 (60.7)	22 (39.3)	
**Duration of chronic illness**							
<5 years	220 (84.9)	12 (4.6)	27 (10.4)	0.239^‡^	161 (62.2)	98 (37.8)	0.281
5 to 10 years	272 (86.9)	14 (4.5)	27 (8.6)		214 (68.4)	99 (31.6)	
>10 years	71 (80.7)	2 (2.3)	15 (17.0)		56 (63.6)	32 (36.4)	
**Annual hospital visit frequency**							
1 to 5 times	312 (87.2)	19 (5.3)	27 (7.5)	**0.010-** ^‡^	209 (58.4)	149 (41.6)	**<0.001**
6 to 10 times	103 (86.6)	5 (4.2)	11 (9.2)		75 (63.0)	44 (37.0)	
>10 times	148 (82.0)	4 (2.2)	31 (16.9)		147 (80.3)	36 (19.7)	
**Forms of tobacco**							
^a^Smoked tobacco	65 (81.3)	5 (6.3)	10 (12.5)	0.250	53 (66.3)	27 (33.8)	0.105
^b^Smokeless tobacco	433 (86.9)	17 (3.4)	48 (9.6)		333(66.9)	165 (33.1)	
Both	65 (79.3)	6 (7.3)	11 (13.4)		45 (54.9)	37 (45.1)	
**Daily tobacco use frequency**							
1 to 5 times	358 (87.1)	14 (3.4)	39 (9.5)	0.204	280 (68.1)	131 (31.9)	**0.004**
6 to 10 times	128 (84.2)	6 (3.9)	18 (11.8)		102 (67.1)	50 (32.9)	
>10 times	77 (79.4)	8 (8.2)	12 (12.4)		49 (50.5)	48 (49.5)	
**Time to first tobacco use of the day**							
Up to 30 minutes after waking up	139 (84.2)	11 (6.7)	15 (9.1)	0.188	102 (61.8)	63 (38.2)	0.555
31 to 60 minutes after waking up	162 (84.8)	10 (5.2)	19 (9.9)		127 (66.5)	64 (33.5)	
Beyond 60 minutes after waking up	262 (86.2)	7 (2.3)	35 (11.5)		202 (66.4)	102 (33.6)	
**Duration of tobacco use**							
≤10 years	118 (90.8)	1 (0.8)	11 (8.5)	**0.026** ^‡^	93 (71.5)	37 (28.5)	0.185
11 to 20 years	152 (88.9)	6 (3.5)	13 (7.6)		113 (66.1)	58 (33.9)	
>20 years	293 (81.6)	21 (5.8)	45 (12.5)		225 (62.7)	134 (37.3)	
**Overall knowledge**							
No	NA	NA	NA	NA	350 (62.2)	213 (37.8)	**<0.001**
Low	NA	NA	NA	NA	21 (75.0)	7 (25.0)	
Moderate/High	NA	NA	NA	NA	60 (87.0)	9 (13.0)	

*Notes***:** ^**a**^Smoked tobacco included bidis and cigarettes; ^b^Smokeless tobacco comprised gul, zarda, and sada pata; ^†^Pearson’s chi-square test; ^‡^Fisher’s exact test; NA, Not applicable; Bold values indicate significant results.

### 3.5 Factors associated with overall knowledge of and willingness to use cessation services

Table 4 summarises the results of the multivariable analyses examining the factors associated with overall knowledge of cessation services and willingness to use cessation services. In the multivariable ordinal logistic regression analysis with partial proportional odds, Participants who had completed secondary education and those with higher secondary education or above had 1.9 times (95% CI: 1.08–3.31) and 4.3 times, respectively (95% CI: 2.21–8.51) higher odds, respectively, of possessing overall knowledge of cessation services compared to individuals who were illiterate or had completed primary education.

**Table 4:** Multivariable analyses of factors associated with overall knowledge of and willingness to use cessation services among study participants.

Variable	^a^Overall knowledge of cessation services			^b^Willingness to use cessation services		
	AOR	95% CI	p-value	AOR	95% CI	p-value
**Sex**						
Male	Reference			Reference		
Female	0.6	0.26 to 1.33	0.201	0.8	0.40 to 1.48	0.447
**Education level**						
Illiterate/Primary	Reference			Reference		
Secondary	1.9	1.08 to 3.31	0.025*	1.8	1.12 to 2.83	0.016*
Higher secondary and above	4.3	2.21 to 8.51	<0.001***	2.2	1.13 to 4.68	0.026*
**Occupational category**						
Housewife/Unemployed	Reference			Reference		
Businessperson	1.4	0.52 to 3.53	0.532	0.5	0.22 to-1.09	0.084
Service holder	1.1	0.39 to 2.88	0.908	0.5	0.20 to 1.15	0.97
Manual worker	0.7	0.26 to1.77	0.435	0.4	0.21 to 0.86	0.017*
**Monthly family income**						
<20,000 BDT	Reference			NI	NI	NI
20,000–30,000 BDT	0.9	0.53 to 1.51	0.664	NI	NI	NI
>30,000 BDT	1.5	0.75 to 3.01	0.248	NI	NI	NI
**Annual hospital visit frequency**						
≤ 5 times	---	---	---	Reference		
6 to 10 times	---	---	---	1.1	0.71 to 1.76	0.633
>10 times	---	---	---	2.5	1.59 to 3.88	<0.001***
**Duration of tobacco use**						
≤10 years	Reference			NI	NI	NI
11 to 20 years	1.3	0.61 to 2.95	0.473	NI	NI	NI
>20 years	2.0	0.98 to 3.99	0.055	NI	NI	NI
**Overall knowledge of cessation services**						
No	NA	NA	NA	Reference		
Low	NA	NA	NA	2.2	0.89 to 5.88	0.103
Moderate/High	NA	NA	NA	3.5	1.73 to 7.99	0.001**

*Notes***:** ^a^Adjusted for sex, education level, occupational category, monthly family income, annual hospital visit frequency, and duration of tobacco use; ^b^Adjusted for sex, education level, occupational category, annual hospital visit frequency, daily tobacco use frequency, and overall knowledge of cessation services; aOR, Adjusted odds ratio; CI, Confidence interval; NI, Not included in the multivariable logistic regression model; ---Proportional odds assumption was violated, and so no common OR was estimated by default in R software; NA, Not applicable**;** *p < 0.05; **p < 0.01; ***p < 0.001**.**

The multivariable binary logistic regression analysis showed that participants with secondary education, as well as those with higher secondary and above education were 1.8 times (95% CI: 1.12–2.83) and 2.2 times (95% CI: 1.13–4.68) more likely, respectively to express willingness to use cessation services compared to participants who were illiterate or had completed primary education. Manual workers were 60% less likely to report willingness to use cessation services than housewives or unemployed participants (95% CI: 0.21–0.86). Participants who had made more than 10 hospital visits in the 12 months preceding the study had 2.5 times higher odds of being willing to use cessation services compared to those who had made five or fewer visits during the same period (95% CI: 1.59–3.88). In addition, participants with moderate to high levels of overall knowledge of cessation services were 3.5 times more likely to express willingness to use such services than those with no knowledge of cessation services (95% CI: 1.73–7.99).

## 4 Discussion

### 4.1 Major findings of the study

To the best of the authors’ knowledge, this is the first study in Bangladesh to examine the factors associated with overall knowledge of and willingness to use cessation services among patients with NCDs. The multivariable analyses identified education level as the only significant predictor of overall knowledge of cessation services. Similarly, willingness to use cessation services was significantly associated with education level, occupational category, annual hospital visit frequency, and overall knowledge of cessation services. These findings provide valuable evidence to guide the development of context-specific interventions aimed increasing awareness of and uptake of cessation services, thereby supporting tobacco cessation and sustained abstinence.

#### 4.1.1 Outcome one: Overall knowledge of cessation services.

Only a small proportion (10.5%) of participants in this study reported moderate to high levels of overall knowledge about tobacco cessation services, highlighting a significant awareness gap in patients with NCDS, a high-risk population. This limited knowledge of cessation services may be attributable to health system and provider-related barriers. In Bangladesh, cessation services are not routinely offered or are inconsistently available in most healthcare settings [18,33], resulting in fewer opportunities for patients to become aware of them through routine clinical encounters. Clinical priorities may also constrain the delivery of cessation services. Healthcare providers often prioritise immediate disease management and treatment over behavioural counselling due to high patient loads [34], limited consultation time [35], and the absence of standardised protocols, which may limit patients’ exposure to cessation information. Another contributing factor may be inadequate knowledge and skills among healthcare providers regarding tobacco cessation in Bangladesh [35,36]. A lack of training can lead to low provider confidence, insufficient counselling skills, and poor awareness of available cessation resources. As a result, providers may be less likely to initiate discussions on tobacco use or to inform patients about available cessation services, thereby indirectly contributing to patients’ low awareness and knowledge. To address these gaps, strengthening tobacco cessation services in Bangladesh requires integrating standardised cessation protocols into routine NCD care and ensuring their consistent availability across all levels of healthcare. Furthermore, targeted training and capacity-building for healthcare providers should be prioritised to improve counselling skills, confidence, and awareness of cessation resources.

The results of this study showed that higher educational attainment was significantly associated with greater knowledge of cessation services. This association could be attributable to greater health literacy among individuals with higher education [37], as education improves the capacity to acquire, process, and apply health information. Higher educational attainment is also linked to better cognitive skills [38], which may support more effective navigation of health systems and interpretation of cessation-related messages. In addition, educational differences may operate through unequal access to information channels. People with higher levels of education are more likely to access mass media [39,40] and digital platforms [41], which are important sources of health information, particularly in LMIC settings. However, this reflects underlying structural inequalities, whereby less-educated people face compounded barriers to accessing and utilising health information. These findings suggest that improving knowledge of cessation services requires not only increasing information availability but also addressing disparities in how information is communicated and accessed. Tailored communication strategies such as the use of simplified messaging, culturally appropriate audio-visual materials, and community-based outreach may be important for reaching people with lower levels of education.

#### 4.1.2 Outcome two: Willingness to use cessation services.

In this study, the majority of participants (65.3%) reported willingness to use cessation services, suggesting substantial latent demand for cessation support among patients with NCDs in Bangladesh. This finding is important because it suggests that reluctance quit tobacco use is not the primary barrier in this population; rather, the main challenge lies in translating positive intention into actual service uptake within an enabling health system environment. A plausible explanation for the greater willingness is growing awareness of the harmful health effects of tobacco use, particularly among individuals already living with NCDs. The experience of chronic illness may heighten risk perception and increase motivation to modify health behaviours [42], thereby strengthening intentions to seek cessation support. However, from a behavioural perspective, intention alone is not sufficient to drive action. The intention-behaviour gap indicates that even strong motivation may not lead to service uptake when structural and contextual barriers are present. In the Bangladeshi context, several system-level constraints may limit the translation of willingness into utilisation. Tobacco cessation services are not consistently integrated into routine NCD care, resulting in missed opportunities for provider-initiated counselling and referral. Consequently, patients may have limited awareness of where and how to access cessation support, even if they are motivated to quit. In addition, the absence of standardised cessation pathways within primary healthcare settings reduces service visibility and weakens continuity of care for tobacco dependence treatment. These findings highlight the need to strengthen the integration of tobacco cessation services into routine NCD care, ensuring that brief counselling and clear referral pathways are systematically delivered at every patient encounter. In addition, capacity-building for healthcare providers and the establishment of standardized, easily accessible cessation protocols within primary healthcare settings are essential to translate existing willingness into actual service uptake.

This study revealed that higher educational attainment was significantly associated with greater willingness to use cessation services. This observation aligns with findings from a US study among smokers, which reported lower willingness to engage in counselling individuals with a high school education or less compared to those with higher education [43]. A plausible explanation is that individuals with higher education may have a better understanding of cessation benefits, greater ability to navigate available services, and improved communication with healthcare providers, which can enhance motivation to seek cessation support. However, willingness alone may not be sufficient to ensure actual utilisation of cessation services. This gap may be influenced by several factors, including perceptions of the effectiveness of cessation support, social norms surrounding tobacco use, concerns about cost or accessibility, and the limited availability of services in healthcare facilities. These barriers are more likely to be more pronounced among individuals with lower educational attainment, who may also have lower trust in formal health services and reduced confidence in their ability to quit. These findings suggest that improving willingness requires not only increasing awareness but also addressing key perceptual and structural barriers. Efforts should therefore focus on improving the availability and accessibility of cessation services, reducing cost-related barriers, and delivering clear, culturally appropriate information to enhance trust and engagement, particularly among less-educated populations.

According to the findings of this study, manual workers were less willing to use cessation services. The observation could be partly explained from an economic perspective. Manual workers usually have lower socioeconomic status [44,45], which reduce ability to access formal cessation services [46]. Limited access to these services may, in turn, reduce their exposure to cessation information and counselling, thereby lowering willingness to use cessation services. In addition, tobacco use in workplace environments in Bangladesh, where manual workers are employed is normalized, which may decrease motivation to seek professional cessation support. These findings highlight the need for targeted, workplace-based cessation interventions for manual workers. Integrating brief counselling and awareness activities into occupational health and workplace settings could improve reach and engagement. Subsidised or low-cost cessation support, including pharmacotherapy where appropriate, may help reduce financial barriers. Strengthening enforcement of smoke-free workplace policies and implementing tailored health education campaigns addressing social norms around tobacco use in manual labour settings are also recommended to enhance quitting motivation and increase utilisation of cessation services.

In this study, greater knowledge of tobacco cessation services was significantly associated with higher willingness to use such services compared to no knowledge. This finding is consistent with evidence from Saudi Arabia, where awareness has been shown to increase utilisation of cessation services [47]. Although knowledge is an important enabling factor that helps individuals understand where and how to access cessation support, its effect is strongly shaped by the health system context in which services delivered. In Bangladesh, the lack of integration of cessation services into primary care and NCD management, along with inconsistent delivery, reduces opportunities for healthcare providers to routinely screen, counsel, and refer patients for cessation support. Consequently, even knowledgeable patients may lack clear and accessible pathways to cessation support. On the other hand, in more resource-advantaged settings, awareness is more likely to translate into service use due to the ready availability of structured cessation services. These findings highlight the need to strengthen the integration of cessation services into routine primary and NCD care in Bangladesh to ensure consistent delivery. Healthcare providers should be trained to routinely deliver brief advice and referrals during consultations. In addition, establishing clear and standardised referral pathways, including national quitline services, is essential to improve and translate knowledge into actual service utilisation.

### 4.2 Implications of the study

The findings of this study have important implications for strengthening the integration of tobacco cessation services into NCD corners in Bangladesh. The low level of knowledge and greater willingness to use cessation services observed among patients with NCDs indicate missed opportunities within routine care. Integrating structured cessation support, including brief advice, counselling, and referral mechanisms, into NCD corners could improve the reach and uptake of these services among high-risk populations. Given that Upazila Health Complexes serve as the primary point of care for many patients, such integration offers a practical platform for early identification of tobacco users and timely intervention.

The study also highlights the need to strengthen provider capacity through training and the development of standardised guidelines to ensure consistent delivery of cessation support. Enhancing patient awareness through routine counselling and health education within NCD services may further improve utilisation. From a health system perspective, leveraging existing infrastructure and workforce within NCD corners can enhance efficiency without requiring substantial additional resources. However, effective implementation will require supportive policies, monitoring mechanisms, and integration into national tobacco control strategies. Overall, integrating cessation services into NCD corners represents a feasible and scalable approach to reducing tobacco use and improving NCD outcomes in Bangladesh.

### 4.3 Strengths and limitations

A major strength of this study is its focus on patients with NCDs, a group that is particularly vulnerable to the adverse health sequences of tobacco use. By examining both knowledge of and willingness to use tobacco cessation services among this high-risk group, this study addresses an important gap with direct implications for public health policy and clinical practice in Bangladesh. In addition, the relatively large sample size enhances the statistical power of the analyses and increases the precision and reliability of the findings. However, the study has several limitations. First, the cross-sectional design of this study does not permit the establishment of causal relationships between the variables examined [48]. The observed associations may be influenced by unmeasured confounding factors and should therefore be interpreted as indicative rather than causal. Longitudinal studies are needed to establish temporal relationships and to assess whether increased knowledge leads to sustained utilisation of cessation services. Second, the study relied on self-reported data, which may be subject to recall and social desirability bias. Participants may have overreported their knowledge or willingness to use cessation services, potentially leading to overestimation of the observed associations. Third, the use of convenience and purposive sampling at the district and sub-district levels limits the probabilistic nature of the sampling design and may introduce selection bias, although consecutive sampling of participants within facilities may have reduced this risk. Finally, data were collected only from government sub-district hospitals in Bangladesh, which may limit the generalisability of the findings to to other healthcare settings, such as private facilities or tertiary government hospitals, where patient profiles and service delivery contexts may differ. Overall, these limitations should be considered when interpreting the results.

## 5 Conclusions

This study found that knowledge of tobacco cessation services among patients with NCDs in Bangladesh was markedly low, despite a relatively high level of willingness to use such services. Educational attainment emerged as a key determinant of both knowledge and willingness, while more frequent healthcare visits and greater knowledge were positively associated with willingness to use cessation services. Furthermore, engagement in manual occupations was negatively associated with willingness to use cessation services. These findings highlight the urgent need for targeted interventions, including integrating cessation support into routine NCD care, strengthening provider capacity to deliver brief interventions, and ensuring the availability of accessible and standardised pathways, including community-based programmes and quitline support.

## Supporting information

S1 File Survey questionnaire. (DOCX)

S2 File Dataset. (XLSX)
