# Supplementary material for: Knowledge of and willingness to use tobacco cessation services among patients with non-communicable diseases in Bangladesh: A cross-sectional study
Source: PLoS One. 2026 Aug 26;21(8):e0356915. doi: 10.1371/journal.pone.0356915 (PMC13513993; doi:10.1371/journal.pone.0356915)
Supplement: Supplementary file 1 [file pone.0356915.s001.docx]

**Survey Questionnaire**

| **Demographic Characteristics** | | |
| --- | --- | --- |
| **Sl** | **Questions** | **Code number and response options** |
| 1 | Patient’s sex | 1=Male 2=Female |
| 2 | How old are you? | 1=<40 years  2=40 to 59 years  3=≥60 years |
| 3 | What is your educational qualification? | 1= Illiterate/Primary  2=Secondary  3=Higher secondary and above |
| 4 | What is your marital status? | 1= Married  99=Other (Single and widowed) |
| 5 | What is your occupation? | 1=Service holder  2=Businessperson  3=Housewife/Unemployed  4=Manual worker |
| 6 | What is your monthly family income? | 1=<20,000 BDT  2=20,000 to 30,000 BDT  3=>30,000 BDT |
| **Clinical Characteristics** | | |
| 7 | What chronic disease(s) have you have diagnosed with? (Multiple response) | Disease \| Yes=1 \| No=0; Asthma \| 1 \| 0; Bronchitis \| 1 \| 0; COPD \| 1 \| 0; Diabetes \| 1 \| 0; Hypertension \| 1 \| 0; Heart disease \| 1 \| 0; Stroke \| 1 \| 0; CKD \| 1 \| 0; Others \| 1 \| 0; Please specify_______________ \| \| |
| 8 | How many chronic diseases are you suffering from? | 1=1Disease  2=2 diseases  3=≥3 diseases |
| 9 | For how long have you been diagnosed with a chronic illness? | 1=<5 years  2=5 to 10 years  3=>10 years |
| 10 | In the past 12 months, how many times did you visit any health facility for your chronic illness? | 1=1 to 5 times  2=6 to 10 times  3=>10 times |
| **Tobacco Use Patterns** | | |
| 11 | What type of tobacco products do you use? | 1=Smoked tobacco  2=Smokeless tobacco  3=Both |
| 12 | How many times do you consume tobacco each day? | 1=1 to 5 times  2=6 to 10 times  3=>10 times |
| 13 | How soon after you wake up, do you use your first tobacco product? | 1=Within 30 minutes after waking up  2= 31 to 60 minutes after waking up  3= Beyond 60 minutes after waking up |
| 14 | For how many years have you been consuming tobacco? | 1=≤10 years  2=11 to 20 years  3=>20 years |
| **Knowledge of and Willingness to Use cessation Services** | | |
| 15 | Have you ever heard of any tobacco cessation services? | 1=Yes  0=No |
| 16 | If yes, which of the following services have you heard of? (Multiple response) | Service \| 1=Yes \| 0=No; Brief advice \| 1 \| 0; Counselling \| 1 \| 0; Nicotine gum \| 1 \| 0; Nicotine patch \| 1 \| 0; Nicotine logenze \| 1 \| 0; Mouth spray \| 1 \| 0; Medicine \| 1 \| 0; Others \| 1 \| 0; Please specify_________________ \| \| |
| 17 | From which of the following facilities or places can you access tobacco cessation services? (Multiple response) | Facility \| 1=Yes \| No=0; Pharmacy \| 1 \| 0; Private hospital \| 1 \| 0; Government hospital \| 1 \| 0; NGO health centre \| 1 \| 0; Online support \| 1 \| 0; I don’t know \| 1 \| 0; Others \| 1 \| 0; Please specify________________ \| \| |
| 18 | Are you willing to use any tobacco cessation service that can help you to quit? | 1=Yes  0=No |
